# Planning for and responding to pandemic influenza emergencies: it’s time to listen to, prioritize and privilege Aboriginal perspectives

**DOI:** 10.5365/wpsar.2018.9.5.005

**Published:** 2018-11-06

**Authors:** Kristy Crooks, Peter D Massey, Kylie Taylor, Adrian Miller, Sandra Campbell, Ross Andrews

**Affiliations:** a Hunter New England Local Health District, Population Health, Wallsend, New South Wales, Australia.; b College of Medicine and Dentistry, James Cook University, Cairns, Queensland, Australia.; c Hunter New England Local Health District, Population Health, Tamworth, New South Wales, Australia.; d Menzies School of Health Research, Charles Darwin University, Casuarina, Darwin, Northern Territory, Australia.; e Office of Indigenous Engagement, Central Queensland University, Townsville, Queensland, Australia.; f Indigenous Health, Research Division, Central Queensland University, Cairns, Queensland, Australia.; g National Centre for Epidemiology & Population Health, Australian National University, Canberra, Australian Capital Territory, Australia.

Australia’s Indigenous peoples account for 3% of the country’s population yet continue to experience disproportionately higher rates of mortality and hospitalization for many infectious diseases. (*1*) The 2009 influenza pandemic had an inequitable impact on Indigenous peoples in Australia, (*2*) New Zealand, (*3*) the Americas and the Pacific. (*4*) Genuine and tangible actions that include Indigenous peoples in the planning and response for pandemic influenza is overdue. This paper will identify some of the strategies to incorporate the perspectives of Australia’s Indigenous peoples (hereafter Aboriginal) in planning and responding to infectious disease emergencies.

Historically, infectious diseases have had a major impact on Indigenous peoples internationally. In North America, European contact and ensuing economic developments changed the nature of infectious disease ecology and exacerbated the frequency and severity of the problem for this population. (*5*) The European invasion of Australia brought new diseases such as varicella, smallpox, influenza and measles to which Aboriginal people had little or no immunity. (*6*) The influenza pandemic of 1918–1919 had a devastating impact on the Aboriginal population; (*7*) however, the full impact is unlikely to be known because many Aboriginal deaths went unrecorded. (*6*) In the 2009 Australian influenza pandemic, the rate ratio comparing Aboriginal people in New South Wales with non-Aboriginal people was 4.2 for hospital admissions, 3.9 for intensive care unit admissions and 5.6 for deaths. (*8*)

The health science field, dominated by scientific quantitative methods often fails to recognize Aboriginal perspectives (*9*) as Aboriginal ways of knowing and being are fundamentally different and culturally specific. These differences need to be acknowledged and understood by public health professionals and policy-makers and incorporated into health practice and policy. The omission of Aboriginal people from Australia’s pre-2009 pandemic plan (*10*) is an example of how Aboriginal people have been excluded from the planning and response to infectious disease emergencies. While the current Australian pandemic plan highlights the need for equity and two-way communication with Aboriginal people, there are no recommendations on how to achieve this, and, therefore, the plan inadequately addresses the needs of Aboriginal communities. (*11*)

Aboriginal people continue to be the subject of health service delivery and policy without the opportunity to be part of the decision-making about their health. (*12*) Given the historical factors and complexities of contemporary Aboriginal health, a one-size-fits-all approach to pandemic influenza is unlikely to work. (*13*-*15*) Measures to reduce the risk of public health emergencies in Aboriginal communities need to be developed with and led by communities to maximize their acceptance, impact and effect. There must be a clear process of engagement and two-way respectful and meaningful communication with Aboriginal communities to identify culturally appropriate and effective public health control strategies. (*13*)

To ensure cultural appropriateness in pandemic influenza planning and response, management plans and control strategies must appropriately reflect and prioritize the social realities of Aboriginal communities. Families are an intrinsic element in Aboriginal culture; therefore, emphasis on the value of kinship, family structures and social connectedness with a family-centred approach should be adopted. (*13*) Additionally, pandemic influenza control strategies often include household contacts, but this may or may not encapsulate the risk for Aboriginal families where shared lives and communities are different from mainstream Australia. These differences must be incorporated into pandemic influenza planning so that Aboriginal people are no longer disproportionately affected.

Participatory approaches with Aboriginal communities are becoming a more culturally appropriate and acceptable method for strengthening engagement and building community empowerment. (*16*) Collaborative engagement processes using qualitative approaches could provide insight into the diverse community perspectives, (*16*, *17*) and identify barriers to implementation of disease control strategies. (*18*) Plans and control strategies need to:

• be developed early with Aboriginal organizations and key stakeholders;

• be flexible to meet local priorities;

• include how to reduce risk in families and at large community events;

• ensure targeted communication strategies are co-developed;

• have flexible models of health care to access vaccinations and other medical interventions, and

• include a stakeholder engagement plan

Including these aspects in pandemic planning are integral to enable Aboriginal people to achieve the level of risk of influenza as the general population and look to a future where Aboriginal people can thrive.

In this period, before the next influenza pandemic, it is the time to listen, prioritize and privilege Indigenous voices internationally. To privilege Aboriginal voices means more than just an equity approach, it is about removing paternalistic approaches to health care and moving beyond listening to and consulting with Aboriginal people about health issues. It is about creating a space where Aboriginal people are at the centre, guiding decision-making processes within a culturally appropriate governance structure that is built on the principles of collaboration, power-sharing, transparent communication, mutual accountability and shared responsibility. Infectious disease emergency plans developed without respectful and meaningful engagement is identified as a barrier to acceptance and implementation. (*13*) Specific localized plans for Aboriginal communities are needed (*13*) that are culturally centred, reflect the diverse socio-cultural practices and that can be reassessed and updated in collaboration with public health emergency leaders to meet the changing needs of the community. (*16*) Infectious disease emergency planners must, with Aboriginal peoples, develop a robust understanding of the issues, be culturally safe, appropriate, inclusive and responsive in the development of disease control strategies. This can happen only if public health approaches are developed in partnership with Aboriginal people, not for them. Aboriginal people need to be engaged in the dialogue, leading the way in the construction of knowledge that is supportive of self-determination. Privileging Aboriginal voices will enable culturally informed strategies and may reduce inequity and the risk of pandemic influenza.
